# Introduction to this Special Issue on New Thinking on Psychological Health: Find Purpose and Meaning in Life

**DOI:** 10.3390/ijerph20247168

**Published:** 2023-12-12

**Authors:** Carol D. Ryff, Andrew Soren

**Affiliations:** 1 Institute on Aging, MIDUS, University of Wisconsin-Madison, Madison, WI 53706, USA; 2 Eudaimonic by Design, Halifax, NS B3H 3L7, Canada; andrew.soren@eudaimonicbydesign.com

## 1. Introduction

As illustrated by the articles in this Special Issue [1,2], research on meaning and purpose in life has grown exponentially in recent years. Such inquiries offer varying definitions of these constructs, as well as different instruments to empirically assess them. Importantly, a rich array of questions have been asked about these aspects of psychological well-being. For example, from whence do they come? What are early-life influences on the emergence of meaning and purpose? And how are these qualities cultivated over time? Other studies have examined how these constructs are linked with diverse psychosocial factors, including other aspects of well-being (optimism and life satisfaction), mental distress (negative affect and depressive symptoms), social connections (loneliness and social support), and stressful experiences (perceived discrimination and adversity). The contexts surrounding experiences of meaning and purpose have also been examined—specifically, how do work and family life matter with regard to meaning and purpose? Do encounters with nature contribute to these aspects of well-being? What are the cultural angles on these topics? And how might historical and societal contexts matter, such as the outbreak of COVID-19 or civil unrest?

Among the most highly visible, widely cited studies are those linking meaning and purpose to specific aspects of health, such as the risk of various disease outcomes, as well as length of life (mortality). These inquiries brought gravitas to topics seen by some as soft and subjective rather than hard biomedical facts. The breakthrough was the observation that both matter in understanding how lives unfold. Building on these advances, there have also been efforts to translate the science on meaning and purpose into the public square where all can partake.

Importantly, contributions to this Special Issue offer new directions for all the above topics. Below, we provide a brief description of each contribution, framed in terms of the organizational framework sketched above.

**Contributions related to Early Life and the Cultivation of Meaning and Purpose:**

- Dameron, E.; Goeke-Morey, M. The Relationship between Meaning in Life and the Childhood Family Environment among Emerging Adults. *Int. J. Environ. Res. Public Health* **2023**, *20*, 5945. https://doi.org/10.3390/ijerph20115945.
- André, N.; Baumeister, R. Dysfunctional Schemas from Preadolescence as One Major Avenue by Which Meaning Has Impact on Mental Health. *Int. J. Environ. Res. Public Health* **2023**, *20*, 6225. https://doi.org/10.3390/ijerph20136225.
- Ruini, C.; Albieri, E.; Ottolini, F.; Vescovelli, F. Improving Purpose in Life in School Settings. *Int. J. Environ. Res. Public Health* **2023**, *20*, 6772. https://doi.org/10.3390/ijerph20186772.
- Dyrdal, G.; Løvoll, H. Windjammer: Finding Purpose and Meaning on a Tall Ship Adventure. *Soc. Sci.* **2023**, *12*, 459. https://doi.org/10.3390/socsci12080459.

Emily Dameron and Marcie Goeke-Morey consider the impact of the childhood family environment on reported meaning in life among emerging adults. Their inquiry is rooted in attachment theory and the cultivation of belonging in early childhood, thus bringing a rich literature about cold and risky family environments compared to warm and secure environments. They ask what these varied childhood environments mean for mental health in adulthood, noting that mental health is typically conceptualized as the absence of negative symptoms and psychopathology rather than the presence of positive aspects of mental health. A unique feature is the focus on perceived loneliness as a possible mediator of the link between the childhood family environment and meaning in emerging adulthood. Using a sample of 507 students from a private religious university in the U.S., they collected various measures of the childhood family environment, as well as perceived loneliness and meaning in life. Their findings showed that the quality of relationships in childhood, especially with supportive and loving parents, had a strong impact on the sense of meaning in early adulthood. Further, this effect was mediated by loneliness (after controlling for religiosity). A lack of meaning in early adulthood was thus tied to reports of emotionally cold and rejecting family environments through current experiences of feeling left out and isolated from others. The authors pose numerous useful questions for future research and consider implications for clinical practice.

Nathalie André and Roy Baumeister examine how the formation of interpersonal schemas developed during preadolescence may become a major avenue through which meaning impacts mental health in adulthood. They argue that young people living in stressful or hurtful environments may form atypical schemas that help them survive, but later produce problems in forming adult relationships. Multiple case studies illustrating these processes are provided. One child learned to cope by withdrawing from social interaction and excelling in schoolwork; another by denying her own needs and sacrificing herself for the welfare of others; and a third by distrusting others and becoming assertively independent. Each of these rigid schemas that emerged from a need to cope with inter-personal adversity as a child had a destructive impact on adult relationships. The central message is that meaning needs to be approached not only as responses to structured scales aiming to quantify one’s sense of meaning in life but also via schemas that encompass beliefs and concepts that help one to understand the self and the world. Schemas thus enable people to organize and represent their experiences, and in so doing, they can be adaptive or maladaptive. The authors give notable attention to how clinicians can help guide the reinterpretation and restructuring of such schemas to foster behavior change and improve mental health.

Chiara Ruini, Elisa Albieri, Fedra Ottolini, and Francesca Vescovelli explore whether purpose in life can be improved among 614 school children in Northern Italy. In control-based studies, middle and high school students participated in an innovative well-being therapy protocol, while elementary students received a narrative intervention based on fairytales. All students were assessed before and after the interventions on scales of eudaimonic well-being and measures of anxiety and depression. The findings showed that, for both elementary and high school students, purpose in life following the intervention was predicted by initial depressive symptoms and by participation in the intervention vs. the control group. For older students, purpose in life was also predicted by anxiety levels and being female. The overarching objective of this work is to help students develop skills and capacities to set meaningful goals in life while also acknowledging that depressive symptoms and anxiety can be obstacles to the development of purpose in life. The authors encourage policy makers and educational systems to incorporate interventions like these into curricula.

Gunvor Dyrdal and Helga Løoll deconstruct a Norwegian tall-ship adventure, called Windjammer, as a site of meaning making and narrative identity for adolescents who experience social exclusion due to the traumas, challenges, and adversities that they faced growing up. Psychological and demographic data were collected from 122 participants and compared to data from a national adolescent survey. Windjammers experienced life as less meaningful compared to the general population. A four-week adventure was then studied using mixed methods that included a sensory ethnography to generate a deeper understanding of the experiences of three participants. Four themes summarized the adventure: commitment, social well-being, familiarization with seamanship, and self-acceptance. Overall, the experience was linked with increased meaning in life among these adolescents.

Taken together, these contributions offer new insights and findings about experiences in childhood and adolescence that may nurture or undermine the experience of meaning and purpose. Not only are these findings relevant for early life, but they also carry significance for how early experiences may impact mental health and well-being in adulthood, as well as informing valuable clinical and educational interventions along the way.

**Contributions related to Psychosocial Correlates of Meaning and Purpose:**

- Joshanloo, M.; Blasco-Belled, A. Reciprocal Associations between Depressive Symptoms, Life Satisfaction, and Eudaimonic Well-Being in Older Adults over a 16-Year. *Int. J. Environ. Res. Public Health* **2023**, *20*, 2374. https://doi.org/10.3390/ijerph20032374.
- Russo-Netzer, P.; Tarrasch, R.; Niemiec, R. A Meaningful Synergy: The Integration of Character Strengths and the Three Types of Meaning in Life. *Soc. Sci.* **2023**, *12*, 494. https://doi.org/10.3390/socsci12090494.
- Coelho, A.; Lopes, M.; Barata, M.; Sousa, S.; Goes, M.; Bia, F.; Dias, A.; João, A.; Lusquinhos, L.; Oliveira, H.; et al. Biopsychosocial Factors That Influence the Purpose in Life among Working Adults and Retirees. *Int. J. Environ. Res. Public Health* **2023**, *20*, 5456. https://doi.org/10.3390/ijerph20085456.

Mohsen Joshanloo and Ana Blasco-Belled use the dual-continua model of mental health in their analysis of data from the English Longitudinal Study of Aging (ELSA), involving 17,056 participants (average age = 58.8 at baseline) assessed over multiple waves. The key questions were how hedonic (affect balance and life satisfaction) and eudaimonic well-being (optimal psychological and social functioning) were linked with depressive symptoms across time. Within- and between-person sources of variance were disentangled to examine whether increases or decreases in one variable predicted changes in other variables at the next time point. The findings showed positive reciprocal relationships between life satisfaction and eudaimonic well-being, as well as negative reciprocal relationships between the two aspects of well-being and depressive symptoms. Stated otherwise, the results showed that within-person increases in well-being are followed by future decreases in depressive symptoms, while within-person increases in depressive symptoms are followed by future decreases in well-being. Such findings could be used to inform interventions tailored to participants with pre-existing resources and/or vulnerabilities.

Pninit Russo-Netzer, Ricardo Tarrasch, and Ryan Niemiec examine connections between meaning in life and character strengths. After reviewing findings from prior studies over the past two decades, they conducted a large-scale study with 23,641 participants spanning more than 100 countries. They used the Values in Action (VIA) Inventory to measure 24 character strengths, along with three components of meaning (coherence/comprehension, significance/mattering, and purpose) using the Multidimensional Existential Meaning Scale. Meaning was strongly correlated with all character strengths, with the strongest predictors being hope, spirituality, zest, curiosity, and gratitude. Gender differences and age trends were also evident. Additional findings were examined in qualitative analyses to probe synergies between character strengths and meaning.

Anabela Coelho, Manuel Lopes, Marta Barata, and others examine correlates of purpose in life using a cross-sectional sample of 1330 adults aged 55 to 84 from different regions of Portugal. A key focus was on differences between adults who were working versus those who were retired. The findings showed that purpose in life was positively linked with educational level, stress, spirituality (religion) and optimism, social support, and quality of life for both groups. However, age, marital status, and environmental quality helped explain the purpose in life of retired people, whereas quality of life related to social support explained the purpose in life among working adults. As has been found in other studies, increased age was a negative predictor of purpose in life, even among those still working. They call for greater focus on active and engaged aging to help older adults keep a sense of meaning and purpose.

Together, these studies build on findings from other investigations, some longitudinal, underscoring that qualities such as meaning and purpose have a diverse array of psychosocial and sociodemographic correlates. Thus, there is no single or simple account of how these aspects of positive functioning are linked with numerous other constructs, such as life satisfaction, optimism, depressive symptoms, character strengths, social support, or spirituality. Similarly, how meaning and purpose align with sociodemographic factors (age, educational status, and work status) also varies. Investigating these patterns is thus fundamental to mapping the nomological network of meaning and purpose.

**Contributions related to Contexts of Meaning and Purpose in Life:**

- Soren, A.; Ryff, C. Meaningful Work, Well-Being, and Health: Enacting a Eudaimonic Vision. *Int. J. Environ. Res. Public Health* **2023**, *20*, 6570. https://doi.org/10.3390/ijerph20166570.
- Sun, R.; Lau, E.; Cheung, S.; Chan, C. Meaning in Life, Social Axioms, and Emotional Outcomes during the First Outbreak of COVID-19 in Hong Kong. *Int. J. Environ. Res. Public Health* **2023**, *20*, 6224. https://doi.org/10.3390/ijerph20136224.
- Passmore, H.; Krause, A. The Beyond-Human Natural World: Providing Meaning and Making Meaning. *Int. J. Environ. Res. Public Health* **2023**, *20*, 6170. https://doi.org/10.3390/ijerph20126170.

Andrew Soren and Carol Ryff focus on the work context and how it matters for meaning, well-being, and health. They first review scientific findings from the MIDUS (Midlife in the U.S.) national longitudinal study, most of which examined adverse work conditions as influences on poor health. Next, they look at how the organizational literature has demonstrated both the benefits of meaningful work, as well as its dark sides, including the manipulation and exploitation of meaningful work to increase motivation and commitment. A key theme of their review is the need to see decency as a necessary antecedent and to enrich the vision to meaningful work via Aristotle’s writings about virtue, ethics, and the realization of personal potential. They point to recent workplace trends such as the Great Resignation and Quiet Quitting as proof of why this is important. The review calls attention to societal, organizational, and individual interventions that promote both meaning and self-realization while also prioritizing worker rights, emphasizing the need to tie evidence-based practices together at multiple levels, including public policy, organizational culture, and individual strategies.

Rong-Wei Sun, Esther Lau, Sing-Hang Cheung, and Chi-Keung Chan center their analysis of meaning around the double hit of social unrest and the emergence of COVID-19 in Hong Kong in the first quarter of 2020. Not only do such societal events trigger negative emotions, such as anxiety or depressive symptoms, but they may also consolidate beliefs about oneself (meaning in life) and society (social axioms). Their study included 2031 participants from the Formation and Transformation of Beliefs in Chinese (FTBC) project. Their analyses focused on the mediating and moderating effects of the Meaning in Life Questionnaire, with links between social axioms and negative emotions. The findings showed that associations between cynicism and negative emotions were mediated by a low meaning in life, with similar mediation evident between religiosity and negative emotions. Meaning also moderated the link between social cynicism and emotional outcomes. They considered the implications of their findings for understanding the dynamics of social and personal beliefs in affecting mental health during times of public crisis.

Holli-Anne Passmore and Ashley Krause review an emerging literature that has examined the bidirectional relationship between nature and meaning in life. The natural world is a life context receiving increasing interest in research on well-being and health. They propose that nature helps provide meaning by addressing human needs to find coherence, significance/mattering, and purpose. They also consider how nature enhances one’s experiential appreciation for life, which is a possible fourth aspect of meaning in life. A further emphasis is on nature-based activities that build meaningful lives. They conclude by considering how contemporary threats to nature also constitute threats to meaning in life.

Thus, three notably distinct formulations of context—work, major public stressors, and the natural environment—were considered relevant domains for understanding what nurtures or undermines meaning in life. While each article framed the topic and its links to mental and sometimes physical health in diverse ways, as a tryptic, they illustrate a compounding ecosystem of meaning and purpose at work in our lives, from the self at work to the public sphere and to the planet writ large.

**Contributions Linking Meaning and Purpose in Life to Mortality:**

- Berkowitz, L.; Mateo, C.; Salazar, C.; Samith, B.; Sara, D.; Pinto, V.; Martínez, X.; Calzada, M.; Schultzendorf, A.; Pedrals, N.; et al. Healthy Eating as Potential Mediator of Inverse Association Between Purpose in Life and Waist Circumference: Emerging Evidence from US and Chilean cohorts. *Int. J. Environ. Res. Public Health* **2023**, *20*, 7099. https://doi.org/10.3390/ijerph20237099.
- Hill, P.L.; Rule, P.D.; Wilson, M.E. Does Activism Mean Being Active? Considering the Health Correlates of Activist Purpose. *Soc. Sci.* **2023**, *12*, 425. https://doi.org/10.3390/socsci12080425.
- Boylan, J.; Biggane, C.; Shaffer, J.; Wilson, C.; Vagnini, K.; Masters, K. Do Purpose in Life and Social Support Mediate the Association between Religiousness/Spirituality and Mortality? Evidence from the MIDUS National Sample. *Int. J. Environ. Res. Public Health* **2023**, *20*, 6112. https://doi.org./10.3390/ijerph20126112.
- Friedman, E.; Teas, E. Self-Rated Health and Mortality: Moderation by Purpose in Life. *Int. J. Environ. Res. Public Health* **2023**, *20*, 6171. https://doi.org/10.3390/ijerph20126171.
- Song, J.; Kang, S.; Ryff, C. Unpacking Psychological Vulnerabilities in Deaths of Despair. *Int. J. Environ. Res. Public Health* **2023**, *20*, 6480. https://doi.org/10.3390/ijerph20156480.

Arguably, much of the heightened interest in meaning and purpose revolves around how these subjective experiences matter for different aspects of health, as well as for how long people live. Five contributions to this Special Issue expand these lines of inquiry.

Loni Berkowitz, Mateo Camila, Cristian Salazar, and others bring a focus on health behavior using prior evidence linking purpose in life to reduced risk of obesity-related chronic diseases. Specifically, they focus on whether the link between purpose in life and waist circumference is mediated by healthy eating. Drawing on data from 2060 adults in the MIDUS study, as well as 223 Chilean adults from the CHILEMED study, they assessed dietary quality according to food intake data available in each cohort. The findings showed significant associations of purpose in life with healthy eating and low-fat dietary patterns in both the U.S. and Chilean cohorts, respectively, after adjusting for sociodemographic variables. Further analyses showed that the link between sense of purpose and waist circumference, an indicator of abdominal obesity, is partially mediated by healthier food intake in both samples. They conclude with the observation that interventions aimed at increasing purpose in life may facilitate adherence to better dietary patterns.

Patrick Hill, Payton Rule, and Megan Wilson observe that prior studies linking a sense of purpose to different health variables have failed to consider specific forms of purpose. They call attention to a new construct, namely, activist purpose defined as the commitment to engage in social activism. They conducted a cross-sectional study with 307 U.S. adults (mean age: 38.1) to assess activist purpose, a sense of purpose, health, and health behaviors. They also asked about other purpose orientations (prosocial, occupational, personal recognition, and creative endeavors). Their results showed consistent positive associations between a sense of purpose, self-rated health, and health behaviors. Activist purpose levels were positively associated with a higher engagement in health behaviors but not self-rated health. They discuss whether activist purpose should be viewed as health-promoting and what research is needed to evaluate this claim.

Jennifer Morozink Boylan, Christianne Biggane, Jonathan Shaffer, and colleagues investigate whether purpose in life and social support mediate the association between religiousness/spirituality and mortality. They utilized data from the MIDUS national longitudinal study, beginning with a baseline of 6120 participants, of which 1711 had died at the second wave. Their analyses showed that those who attended religious services had a lower mortality than those who did not and, further, that the composite measure of religiousness and spirituality was also associated with a lower mortality risk after adjusting for other factors. Tests of indirect effects further supported the mediating roles of purpose in life and social support in linking religion/spirituality to mortality. They discuss ways in which religious or spiritual communities may foster these psychosocial mechanisms, thereby contributing to healthier and longer lives.

Elliot Friedman and Elizabeth Teas examine links between self-rated health and mortality in two large national longitudinal studies, namely, MIDUS and the Health and Retirement Study (HRS), which together provide 12- to 14-year follow-up periods for mortality analyses. They build on prior findings showing that purpose in life moderated associations between chronic conditions and biological risk factors (inflammatory markers) by examining the role of purpose in life as a moderation of the relationship between subjective health and mortality. The findings showed that purpose in life and self-rated health were both positively associated with longevity and, further, that purpose in life moderated these relationships. Further analyses examined variability in these associations according to race/ethnicity. Their work concludes with the consideration of growing clinical and community-based interventions showing that purpose in life can be promoted.

Jieun Song, Sohyun Kang, and Carol Ryff bring attention to recent evidence of growing deaths of despair (due to suicide, alcoholism, and drug addictions), noting that these prior findings have not explicitly assessed the idea of despair. They used longitudinal data from MIDUS to investigate whether low levels of eudaimonic and hedonic well-being predict increased risk of deaths due to the aforementioned causes compared to deaths due to cancer or heart disease (after adjusting for sociodemographic factors). They focused on 695 reported deaths that occurred between 2004 and 2022. The findings showed that low levels of purpose in life, positive associations with others, personal growth, and positive affect predicted a significantly greater likelihood of deaths of despair (suicide, addictions, and alcoholism) than deaths due to heart disease. These patterns were prominent among better educated adults, suggesting that not having well-being may heighten vulnerability among privileged segments of society, where socioeconomic advantage is known to predict higher levels of eudaimonic and hedonic well-being.

Taken together, these contributions to this Special Issue bring new angles to multiple issues: how purpose matters for abdominal obesity via its links to healthy eating; how purpose mediates the link between religion/spirituality and mortality; how purpose in life is enacted (activist purpose); how purpose moderates the link between self-rated health and mortality; and, finally, the extent to which deficiencies in various aspects of well-being, including purpose and personal growth, contribute to a heighted risk of death due to suicide, alcoholism, and drug addictions compared to the risk of death from heart disease.

**Translational Science and Community Action:**

- Burrow, A. Beyond Finding Purpose: Motivating a Translational Science of Purpose Acquisition. *Int. J. Environ. Res. Public Health* **2023**, *20*, 6091. https://doi.org/10.3390/ijerph20126091.
- Russo-Netzer, P. Building Bridges, Forging New Frontiers: Meaning-Making in Action. *Soc. Sci.* **2023**, *12*, 574. https://doi.org/10.3390/socsci12100574.

The last two contributions to this Special Issue address the wider import of research on meaning and purpose in life. Anthony Burrow calls for a translational science of purpose acquisition. Given the substantial findings that demonstrate a biopsychosocial relationship between purpose and positive mental and physical health outcomes, much more needs to be done on how to acquire purpose if one does not already have it. For Burrows, this is not only public a public health imperative but also a move towards greater social justice and equity, one that would benefit from a strong reciprocal relationship between research and application. A translational science of purpose acquisition would bridge this gap, and Burrows offers a preliminary roadmap for how researchers and community stakeholders might work together to operationalize the science, collect data, learn about how to effectively implement new findings, and collaboratively influence policy decisions.

Similarly, Pninit Russo-Netzer acknowledges the growth of scientific frameworks and findings about meaning but underscores the need for interventions that bridge the gap between research and application in diverse contexts and community-based settings. She illustrates these ideas using an Israeli initiative called Compass that harnesses the community as a central frame of reference to comprehend not only the self but also people and the world through multiple meaning-making activities. Compass employs an ecosystem approach to explore the relationship between individuals, organizations, and their environments through the following: (1) Socratic questions in the public square; (2) an intergenerational life stories project; (3) the use of literature, arts, and museums as meaning-making sites; and (4) education for meaning. For a topic as complex, multi-faceted, and subjective as meaning, Russo-Netzer shows how a transdisciplinary approach can offer context-sensitive and socially responsible interventions that are applicable to everyday life in general society.

Echoing the concluding thoughts of Russo-Netzer regarding the challenges that people face in their daily lives and in the world surrounding them, these contributions reinforce a social responsibility that researchers and practitioners have to extend the focus of meaning and purpose research beyond the ivory tower, as well as beyond individual well-being interventions, into the public square of our communities for the greater good.

## 2. Reflections and Future Directions

### 2.1. What Have We Learned?

Viewed as a whole, the contributions to this Special Issue underscore the richly generative nature of meaning and purpose as topics of scientific inquiry. Also important to emphasize is the diversity of methods, measures, and samples employed to conduct these endeavors. A take-home message is that there is no single right way to investigate these unique aspects of human functioning; rather, the field is advanced by a multiplicity of approaches.

Specific findings illuminate fundamental questions, such as the origins of meaning and purpose. We learned how early childhood development, especially relationships with significant others, shape beliefs and interpersonal schemas that contribute to the cultivation of meaning and purpose in adult life. Other early-life inquiries focused on educational interventions with school children, as well as sailing adventures for at-risk youth. Another fundamental question is how meaning and purpose relate to other constructs. Here, we learned about how eudaimonic well-being is longitudinally aligned with positive emotion, life satisfaction, and depressive symptoms, while other findings examined connections between meaning in life and character strengths. The correlates of purpose in life among working versus retired adults was examined via assessments of stress, spirituality, optimism, and social support. Collectively, these new findings broaden what is known about the interface of meaning and purpose with other relevant psychological topics.

The focus on the contexts of meaning and purpose highlighted important findings, including how such positive experiences can sometimes become negative. An example is the dark side of meaningful work, wherein meaning can sometimes be manipulated and exploited, thereby calling attention to decency as a critical feature in assessing meaningful work. A further context pertained to how meaning in life mediates or moderates negative emotion in the face of social unrest and the pandemic. Another article examined the natural environment to probe how nature helps humans find coherence, significance, and purpose. These diverse formulations of context offer innovative new directions to prior research on meaning and purpose.

An extensively studied topic in past research has been how meaning and purpose matter for mental and physical health. Here, the contributions to this Special Issue carved new territory by showing how the link between purpose in life and waist circumference (an indicator of abdominal obesity) is mediated by healthy eating. A new construct, activist purpose, defined as the commitment to engage in social activism, was found to be positively associated with good health behaviors. Another inquiry showed that the links between religiousness/spirituality and mortality are mediated by purpose in life and social support, while related work showed that purpose in life moderated relationships between self-rated health and morality. A final inquiry in this section investigated whether low levels of eudaimonic well-being increased the risk of deaths of despair due to suicide, addictions, and alcoholism compared to deaths due to heart disease. Supportive evidence was found for low levels of purpose in life, personal growth, and positive associations with others.

Two contributions complete this Special Issue with a focus on translational science and community action. The central objective of both was to examine how experiences of meaning and purpose can become more widely available to those who may lack such qualities. These ideas are framed as public health imperatives, as well as interventions needed to bridge the gap between research and community-based settings. Multiple venues of action were described to offer context-sensitive, socially responsible interventions that are applicable to everyday life.

### 2.2. Where to Go Next?

Regarding future directions, we conclude with advocacy on two fronts. The first is a call for new thinking to weave topics of meaning and purpose together with concerns about human virtue and ethics. This direction reflects an important extension of where science and practice are now and where they need to go. Put succinctly, the cultivation of greater meaning and purpose not only has the potential to benefit human well-being and public health but may also be a critical pathway to address some of the world’s gravest woes, such as widening inequality, systemic racism, and climate change. We see hints towards such commitments to virtue and ethics in the pages of this Special Issue—on the deck of the Windjammer with disenfranchised youth, in attempts to build more cohesive communities post-pandemic, in organizational research seeking to make more decent for more employees, and in efforts to introduce climate change to the study of nature and how it impacts well-being. Taken together, we observe that the pursuit of meaning and purpose often drives people to reach for a more just and fair world. Even so, almost nowhere do we see researchers actively engage in examining the ethical roots of meaning and purpose; similarly, rarely do practitioners work to connect meaning and morality to their interventions with individuals at school, at work, or in the community.

Second, given the growing evidence of the benefits of meaning and purpose, there must be a greater collaboration between researchers and practitioners going forward. Scientific advances should not be sequestered in ivory towers or scholarly journals but must regularly and rapidly be employed to inform evidence-based interventions to be applied and refined in the real world. Similarly, practitioners constantly encounter new questions and contexts that require further research, but few have access to tools or resources to study the effectiveness of such interventions. Bridging the worlds of research and practice in more effective ways is thus a future imperative. In addition, and building on the first point above, both worlds are enriched by embracing virtue and ethics. Interventions that foster meaning and purpose can be vehicles to enact individual talents and capacities while also serving meaningful goals for the workplace, communities, and society.

We conclude by observing that the philosophical origins of meaning and purpose emanate from core questions about what constitutes a good and ethical life. Such queries have emerged from a multitude of perspectives: Ubuntu, Ikigai, Dharma, Ren, the Medicine Wheel, and many other ethno-cultural traditions the world over. Our own philosophical orientation to meaning and purpose stems first and foremost from Aristotle’s formulation of eudaimonia—the ‘good spirit’ within every individual that calls for activity of the soul in accordance with virtue. At the core of eudaimonia is the belief that, like an acorn in the forest, a person has a purpose to potentiate. The acorn’s potential is to become a mighty oak tree. Similarly, an individual’s purpose is to bring their best self, via continued personal growth and ethical action, into the world. Meaning and purpose are necessary but insufficient for that journey. Without an anchor in virtue and ethics, purpose can be malevolent and even evil. Wise readings of human becoming in developmental, existential, and humanistic literatures tell us that eudaimonic well-being also encompasses attributes such as autonomy, environmental mastery, positive relationships, and self-acceptance. That is to say, eudaimonia, inspired by Aristotle, and enriched by later scholarship, includes an array of noble human qualities, but most especially a moral foundation. These need to be part of understanding what virtuous action means in context. The guiding mantra is that an eudaimonic society is a prosocial society—one that prizes good citizens and the pursuit of more just and more fair systems. These are ideals that we actively embrace on the road ahead.

